# Knowledge and Attitude of Working-Class Women Towards Exclusive Breastfeeding in Kwara State, Nigeria: Implications for Counselling Practice

**DOI:** 10.4314/ejhs.v35i5.7

**Published:** 2025-09

**Authors:** Lateef Omotosho Adegboyega, Ifeoma P Okafor, Falilat Anike Okesina, Samuel Kolawole Ajiboye, Muinat Bolanle Bello

**Affiliations:** 1 Department of Educational Guidance and Counselling, Faculty of Education, University of Ilorin, Ilorin, Nigeria; 2 Department of Social Sciences Education, Faculty of Education, University of Ilorin, Ilorin, Nigeria

**Keywords:** Knowledge, Attitude, Working-Class Women

## Abstract

**Background:**

Breastfeeding has long been recognized across cultures and societies as the optimal method of infant nutrition. This study therefore examined the knowledge and attitudes of working-class women towards exclusive breastfeeding in Kwara State, Nigeria.

**Methods:**

A descriptive survey design was employed. Using a simple random sampling technique, 420 respondents were selected. Data were collected through a structured questionnaire. Descriptive statistics, including mean, rank order, and percentages, were used to answer the research questions, while Analysis of Variance (ANOVA) was applied to test the hypotheses at the 0.05 level of significance.

**Results:**

The findings revealed that working-class women in Kwara State possessed knowledge of exclusive breastfeeding, including its benefits, techniques, and barriers. However, 67.1% of respondents demonstrated a negative attitude toward exclusive breastfeeding. The results further showed no significant differences in knowledge of exclusive breastfeeding based on age or educational qualification. In contrast, significant differences were observed in attitudes toward exclusive breastfeeding according to both age (*F=4.83*, *p<0.05*) and educational qualification (*F=11.33*, *p<0.05*).

**Conclusion:**

Although working-class mothers generally possess adequate knowledge of exclusive breastfeeding, there is a need for targeted educational and training programs to improve their attitudes.

## Introduction

Breastfeeding has been recognized throughout human history as the optimal method of infant nutrition across diverse cultures and societies. This practice is supported by a wealth of scientific evidence highlighting its crucial role in infant survival, growth, and development (1). The natural composition of human milk is uniquely tailored to meet the nutritional needs of infants, offering unparalleled benefits that artificial alternatives cannot replicate. Despite the longstanding acknowledgment of breastfeeding's superiority, the advent of industrialization and the development of breast milk substitutes have led to a gradual shift in infant feeding practices. However, research consistently demonstrates that breastfed infants have a significantly higher chance of survival in the early months of life compared to their non-breastfed counterparts (2). This survival advantage underscores the critical importance of promoting and supporting breastfeeding as a public health priority.

Exclusive breastfeeding (EBF) is defined as providing an infant with only breast milk for the first six months of life, excluding any other food or liquid except for necessary medications, vitamins, or supplements (3). This practice is strongly endorsed by leading health organizations worldwide due to its numerous benefits for both infants and mothers. Breast milk provides all the necessary energy and nutrients required for optimal growth and development during the first six months of life. Its benefits extend well beyond this initial period, continuing to contribute significantly to a child's nutritional needs for up to two years and beyond (4). The World Health Organization (WHO) and the United Nations Children's Fund (UNICEF) recommend exclusive breastfeeding for the first six months, followed by continued breastfeeding with appropriate complementary foods for up to two years or longer (1). The unique composition of breast milk adapts to the changing needs of the growing infant, offering a perfect balance of nutrients, antibodies, and bioactive compounds that support immune development, cognitive function, and overall health (5). Moreover, breastfeeding promotes bonding between mother and child, contributing to the emotional and psychological well-being of both.

The implementation of EBF has significantly reduced newborn mortality rates in underdeveloped nations by preventing diarrhoea and infectious diseases. Additionally, it has been shown to reduce the transmission of HIV from mother to child compared to mixed feeding (6). Despite these benefits, EBF rates vary considerably across regions. In Ethiopia, a study by Tadesse et al. (7) found that only 59.3% of mothers practised EBF for the first six months. This rate is higher than the 37.7% reported by Tadele and Habta (8) but still falls short of optimal levels. In contrast, a study conducted in rural Ghana by Diji et al. (9) revealed a much higher EBF rate of 71.0% among lactating mothers, although this was still lower than the 92.6% reported by Victor et al. (10).

In Nigeria, although the mean duration of breastfeeding has been increasing, EBF and early initiation rates remain low (11). Studies in urban and rural communities in the southeast revealed that only 35.9% and 10.0% of women practised EBF, respectively (12, 13). Another study in Ibadan, Southwest Nigeria, reported that after 12 months of follow-up, none of the babies of urban elite women were breastfed, compared to 100% and 80.8% of babies in the rural poor and urban poor groups, respectively (14). Ignorance, undesirable sociocultural beliefs, and misconceptions prevailing in communities have been reported as major barriers to optimal breastfeeding practices (15, 11).

Knowledge of exclusive breastfeeding plays a pivotal role in promoting its practice among working women. The study by Tadesse et al. (7) in Ethiopia demonstrated that mothers with good knowledge of EBF were more likely to adhere to the practice compared to those with poor knowledge. Well-informed mothers are more likely to recognize EBF's numerous benefits, such as enhanced immune protection, reduced risk of infections, improved cognitive development, and lower risks of chronic diseases later in life (2). For mothers, benefits include reduced risks of breast and ovarian cancers, faster postpartum weight loss, and delayed return of menstruation.

Knowledge about EBF also encompasses breastfeeding techniques, which can help working mothers manage common challenges such as latching difficulties, nipple pain, and maintaining milk supply while away from their infants. A study by Nukpezah et al. (16) found that mothers educated on proper breastfeeding techniques were more likely to continue EBF after returning to work. In addition, working mothers with good knowledge of EBF are more likely to be familiar with milk expression and storage techniques, which are crucial for maintaining EBF during work hours. Bai et al. (17) found that education on milk expression and storage significantly improved EBF rates among working mothers. Awareness also empowers women to advocate for workplace rights, such as breastfeeding breaks or pumping facilities. Murtagh and Moulton (18) highlighted that knowledge of workplace support policies was positively associated with continued breastfeeding among employed mothers. Similarly, education programs addressing cultural myths about breastfeeding were found to significantly improve EBF rates in Ghana (19).

Age and educational attainment also influence knowledge of EBF. In Egypt, Abou-ElWafa and El-Gilany (20) found that older mothers (>30 years) had significantly better knowledge compared to younger mothers, possibly due to greater life experience or previous childbirth. However, Tadesse et al. (7) reported no significant association between maternal age and EBF knowledge in Ethiopia, suggesting that age may not always be a determining factor. Educational qualification, on the other hand, consistently shows a positive correlation. Mogre et al. (21) found that mothers with higher education in Ghana demonstrated better knowledge of EBF, while Maharlouei et al. (22) in Iran reported that women with university education had significantly higher knowledge scores compared to those with lower education. Kasahun et al. (23) further observed that while age alone was not significantly associated with knowledge, older mothers with higher educational attainment demonstrated a better understanding of EBF practices.

Despite the knowledge of working-class women regarding EBF, their attitudes may vary significantly depending on age and educational level. Older women, particularly those with prior breastfeeding experience, tend to hold more positive attitudes towards EBF. A study by Singh et al. (24) found that older working-class women were more confident in their ability to exclusively breastfeed, likely due to experience and exposure to breastfeeding practices. Conversely, younger women, especially first-time mothers, often expressed concerns about the feasibility of EBF, citing limited knowledge and anxiety about balancing work and childcare. According to Akinduro et al. (25), educated working-class women were more likely to initiate and sustain EBF than those with lower educational levels, largely due to better access to information, stronger health literacy, and greater understanding of breastfeeding's long-term benefits. Furthermore, educated women were more likely to seek and utilize breastfeeding support services, such as lactation consultants and breastfeeding-friendly workplaces.

## Methods

**Research design**: The study employed a descriptive survey research design.

**Population and sample**: The study population comprised all working-class women in Kwara State, estimated at 750,674 (National Population Commission, 2023). The target population consisted of literate working-class women. Based on the recommendation of Research Advisors (2006), a sample size of 384 was considered appropriate for a population of this magnitude at a 95% confidence interval and a 2.5% margin of error. To account for possible attrition, 10% was added, resulting in a final sample size of 422 respondents.

**Sampling procedure**: A multistage sampling procedure was adopted:

**Stage One- Selection of local government areas (LGAs)**: A simple random sampling technique was used to select three LGAs from each of the three senatorial districts of Kwara State:

Kwara Central: Asa, Ilorin East, Ilorin South, Ilorin West
Kwara North: Baruten, Edu, Pategi, Kaiama, Moro
Kwara South: Ekiti, Oke-Oro, Offa, Ifelodun, Irepodun, Isin, Oyun

In total, nine LGAs were selected out of the sixteen in the state. The selection was carried out using the dip-hat method, where the names of all LGAs were written on slips of paper, placed in a bag, mixed, and randomly drawn.

**Stage Two- Selection of institutions/locations**: A purposive sampling technique was employed to select institutions and locations where large numbers of literate working-class women could be found, such as schools, mosques, churches, and ministries.

**Stage Three-Selection of respondents**: From the identified institutions/locations, literate working-class women were selected using a simple random sampling method. In total, 420 respondents participated in the study.

**Instrument for data collection**: Data were collected using a structured questionnaire titled “Knowledge and Attitude Towards Exclusive Breastfeeding Questionnaire” (KAEBQ) consisting of 25 items. The instrument's content validity was established through expert review. Reliability was assessed using the test–retest method, which produced coefficients of 0.83 for the knowledge scale and 0.78 for the attitude scale.

**Data analysis**: Descriptive statistics, including frequency counts and percentages, were used to summarize the demographic characteristics of respondents and address the research questions.

Knowledge of EBF (Research Question 1): An aggregate mean score of 2.50 served as the benchmark. Knowledge was assessed across four domains:

General knowledge of EBF
Knowledge of benefits for the mother
Knowledge of barriers to EBF
Knowledge of breastfeeding techniques and practices

Scores above 2.50 indicated good knowledge, while scores below 2.50 indicated poor knowledge.

**Attitude towards EBF (Research Question 2)**: A cut-off score of 37 was established. Respondents scoring 37 and above were classified as having a positive attitude, while those scoring below 37 were classified as having a negative attitude.

Inferential statistics were also applied. Analysis of Variance (ANOVA) was used to test the research hypotheses at a 0.05 level of significance.

**Ethical considerations**: Respondents were provided with an informed consent form attached to the questionnaire. The objectives of the study were explained, and participation was entirely voluntary, with the option to withdraw at any time. Confidentiality and anonymity of responses were assured throughout the process.

## Results

The demographic distribution of respondents (Table 1) is based on 420 participants, focusing on age and educational qualifications. The age distribution shows that the majority of respondents were 26 years and above comprising 42.4% of the sample. There was a fairly even split between those aged 26–30 and those aged 31 and above, while the youngest group (18–25 years) represented the smallest proportion.

**Table 1:** Distribution of the respondents' demographic data (N=420)

Variable	Frequency	Percent
**Age**		
18-25 years	78	18.6
26-30 years	178	42.4
31 years and above	164	39.0
**Educational Qualification**		
SSCE and below	124	29.5
NCE/OND	117	27.9
First degree/HND	153	36.4
Postgraduate	26	6.2

Sig. = Significant; SSCE = Senior School Certificate Examination; NCE/OND = National Certificate in Education/Ordinary National Diploma; HND = Higher National Diploma.

Regarding educational qualifications, respondents reflected a diverse range of attainment levels. The largest group (36.4%) held a first degree or HND, followed by those with SSCE and below (29.5%) and those with NCE/OND (27.9%). Postgraduate degree holders represented the smallest group at 6.2%.

Research Question 1: Knowledge of Working-Class Women Towards Exclusive Breastfeeding in Kwara State.

The mean and rank order (Table 2) indicate that all segments had aggregate mean scores above the 2.50 cut-off point. This suggests that working-class mothers demonstrated good general knowledge of exclusive breastfeeding, including its benefits for mothers, techniques and practices, and barriers.

**Table 2:** Mean and rank order analysis on the knowledge of the respondents about exclusive breastfeeding

As far as I am concerned:	Mean	Aggregate	Rank
***General Knowledge of Exclusive Breastfeeding***			
Exclusive breastfeeding is the best form of nutrition for infants during the first six months of life	3.50		
Exclusive breastfeeding helps in building a strong immune system for the infant	3.41		
A baby should receive only breast milk without any additional foods or drinks, not even water, during the first six months	3.30		
Exclusive breastfeeding can reduce the risk of certain infections in infants	2.57		
Breastfeeding should continue along with appropriate complementary foods after six months	3.37	**3.23**	1^st^
***Knowledge of Benefits for the mother***			
Exclusive breastfeeding can help mothers lose weight gained during pregnancy	3.15		
Breastfeeding exclusively reduces the mother's risk of developing breast and ovarian cancers	3.22		
Exclusive breastfeeding can delay the return of menstrual cycles postpartum	3.25		
Breastfeeding promotes bonding between mother and child	3.09	**3.17**	2^nd^
***Knowledge of Barriers to Exclusive Breastfeeding***			
Returning to work can make it difficult to exclusively breastfeed	3.10		
Lack of support from family members can hinder exclusive breastfeeding	3.20		
Breastfeeding in public places can be challenging for some mothers	2.73	**3.01**	4^th^
***Knowledge of Breastfeeding Techniques and Practices***			
Proper latching is crucial for successful exclusive breastfeeding	3.10		
Frequent breastfeeding helps in maintaining an adequate milk supply	3.10		
Mothers should breastfeed on demand rather than on a strict schedule	3.27	**3.15**	3rd

**Research Question 2**: Attitude of Working-Class Women Towards Exclusive Breastfeeding in Kwara State.

The percentage distribution shows that 67.1% (n=282) of respondents had a negative attitude towards exclusive breastfeeding, while 32.9% (n=138) had a positive attitude.

Four Hypotheses Testing was conducted:

⚬ **Hypothesis One**: No significant difference was found in knowledge based on age (F=1.90, p>0.05).
⚬ **Hypothesis Two**: No significant difference was found in knowledge based on educational qualification (F=1.52, p>0.05).
⚬ **Hypothesis Three**: A significant difference was found in attitude based on age (F=4.83, p<0.05).
⚬ **Hypothesis Four**: A significant difference was found in attitude based on educational qualification (F=l 1.33, p<0.05).

To determine where these differences lay, Scheffe Post-Hoc analysis was conducted (Table 3). The results showed that respondents aged 16–25 and 26–30 years had the highest mean scores (37.15 and 37.65, respectively, in subset 2). In terms of educational qualification, respondents with SSCE and below had the highest mean score of 38.79 (subset 3), contributing to the observed significant differences.

**Table 3:** Scheffe post-hoc where the significant difference lies based on age and educational qualification

	N	Subset 1	Subset 2	Subset 3
**Age**				
31 years and above	164	35.45		
18-25 years	78		37.15*	
26-30 years	178		37.65*	
Sig.		.139	.845	
**Educational Qualification**				
Postgraduate	26	31.00		
NCE/OND	117		35.99	
First degree/HND	153		36.51	
SSCE and below	124			38.79*
Sig.		1.000	.115	1.000

* Contribute to the significant difference

Sig. = Significant; SSCE = Senior School Certificate Examination; NCE/OND = National Certificate in Education/Ordinary National Diploma; HND = Higher National Diploma

## Discussion

The findings revealed that working-class mothers in Kwara State generally possessed knowledge of exclusive breastfeeding, including its benefits, techniques, and barriers. This suggests that public health initiatives, educational programs, and community outreach efforts have successfully communicated the importance of EBF. The result is consistent with Radzyminski and Callister (28), who reported that while many mothers were aware of breastfeeding benefits, they faced challenges in practice, especially when returning to work. Rollins et al. (29) also noted that breastfeeding education and support interventions effectively improve knowledge and initiation rates but highlighted that structural barriers, such as inadequate maternity leave, continue to undermine EBF among working mothers.

Despite this knowledge, 67.1% of respondents demonstrated a negative attitude towards exclusive breastfeeding. Such attitudes may manifest as reluctance to initiate or continue breastfeeding, preference for formula feeding, or early weaning. Contributing factors include the perceived inconvenience of breastfeeding, workplace barriers, cultural beliefs, and insufficient awareness of its benefits. This aligns with findings by Tadesse et al. (7), who reported that maternal employment negatively influences EBF practices. Similarly, Osibogun et al. (15) observed that employed mothers in Nigeria were less likely to exclusively breastfeed compared to unemployed mothers, largely due to work-related barriers. Karimi et al. (30) further reported that inadequate workplace support and limited breastfeeding management knowledge hindered EBF among working mothers.

The study also found no significant difference in knowledge of exclusive breastfeeding based on age or educational qualification. This indicates that both younger and older working-class women, as well as women across education levels, had comparable levels of knowledge. The finding is consistent with Nukpezah et al. (16), who found no significant association between maternal age and EBF in Ghana, and Karimi et al. (30), who reported a similar outcome in Ethiopia. This may reflect widespread dissemination of EBF information through healthcare facilities, community programs, and media campaigns, which reach women regardless of demographic background.

However, attitudes towards EBF did differ significantly by age and education. Younger mothers (16–30 years) were more likely to demonstrate distinct attitudes compared to older women. This finding aligns with Tarrant et al. (31), who reported that younger mothers were more likely to intend to and actually practise EBF than older mothers. In terms of education, respondents with SSCE and below qualifications contributed most to the difference in attitudes. This suggests that lower education levels may correlate with limited awareness, weaker health literacy, and reduced understanding of EBF benefits. Akinduro et al. (14) similarly reported that higher maternal education was associated with better breastfeeding practices in Nigeria.

In conclusion, the study concludes that while working-class women in Kwara State possess adequate knowledge of exclusive breastfeeding, with two-third of women holding negative attitudes towards it. Age and educational qualification were found to influence attitudes but not knowledge. This suggests that factors beyond demographic characteristics such as workplace policies, cultural norms, and support systems play a major role in shaping breastfeeding attitudes and practices.
